# Diagnostic Difficulties and Complexities in the Management of Dermatofibrosarcoma Protuberans in the Breast: A Case Report and Review of the Literature

**DOI:** 10.7759/cureus.17005

**Published:** 2021-08-08

**Authors:** Justin Y Ng, Jessica Y Ng, Kimberley Tan, Rhea Liang

**Affiliations:** 1 General Surgery, Gold Coast University Hospital, Gold Coast, AUS; 2 Surgery, Gold Coast University Hospital, Gold Coast, AUS; 3 Surgery, Robina Hospital, Gold Coast, AUS

**Keywords:** breast, cancer, dermatofibrosarcoma protuberans, surgery, breast benign and malignant surgery

## Abstract

Dermatofibrosarcoma protuberans (DFSP) is an extremely rare sarcoma with an incidence between 0.8-5.0 cases per one million persons per year. DFSP accounts for less than 0.1% of all malignancies and approximately 1-6% of all soft tissue sarcomas. Only a few cases of DFSP have been found within the breast tissue. We report a case of DFSP in a 30-year-old female within the left breast. The sarcoma presented as a painless, rubbery, mobile lump that gradually increased in size. It was initially identified on an ultrasound scan and subsequently confirmed with MRI and core biopsy.

DFSP is a rare condition and treatment guidelines are not well established. The current recommendation is for surgical excision with 2-cm margins. Mastectomy may be considered in some circumstances. In our case, the aim was for surgical resection with 2-cm margins at both breast tissue and skin, but insufficient margins were taken. The recommendation for re-excision of the inadequate margins was declined. It is uncertain what the implications of this are given the lack of research on DFSP in the breast. Therefore, close surgical surveillance will be imperative. We present this case to highlight the difficulties associated with the diagnosis, treatment, and management of DFSP due to the lack of literature on this disease.

## Introduction

Dermatofibrosarcoma protuberans (DFSP) is an extremely rare sarcoma with an incidence between 0.8-5.0 cases per one million persons per year [1-3]. It accounts for less than 0.1% of all malignancies [2] and approximately 1-6% of all soft tissue sarcomas [1,2,4,5]. Only a few cases of DFSP have been found within the breast tissue. 

The current surgical options include either surgical excision with recommended margins of at least 2 cm or mastectomy with or without reconstruction. Selection of either option will require a detailed discussion with the patient.

## Case presentation

A 30-year-old Australian female of Korean descent presented to the general practitioner with a left breast lump. The patient reported that the lump was non-tender, mobile, and had been gradually increasing in size and grown to approximately 1 cm at the time of presentation. The patient had no history of chemical, radiation, or hormonal exposure other than ethinyloestradiol/levonorgestrel, which she had taken for 11 years and had stopped since the diagnosis. The patient had no history of any surgeries or breast trauma and there was no family history of any breast cancer or DFSP. She had consumed one glass of wine weekly for the past five to six years, was a non-smoker, and had no history of recreational drug use.

On examination, the patient was slim, with a body mass index of 19.1 and size B breasts. The breast lump was palpable at the lateral margin of the left breast at 3 O’clock, 2 x 3 cm in size, mobile and non-tender with no skin discoloration or tethering. Examination of the contralateral breast was normal. No axillary or supraclavicular nodes were identified, and the liver was not palpable.

**Figure 1:**
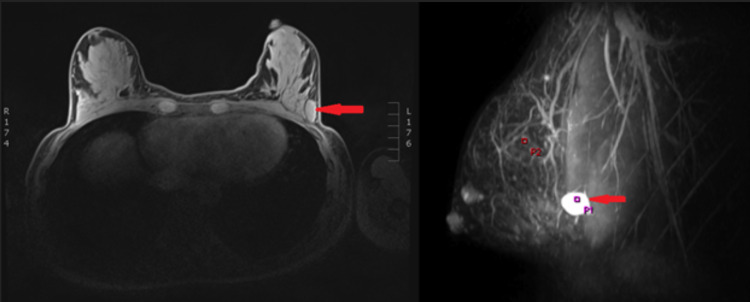
Bilateral MRI breast demonstrating the breast DFSP MRI demonstrated a predominantly benign enhancement; however, the central portion showed an area of increased vascularity with a very rapid enhancement kinetic curve. The lesion was seen in the subcutaneous tissue, superficial to the pectoral fascia, and the mass exhibited a high signal intensity on the T2-weighted images (arrows) MRI: magnetic resonance imaging; DFSP: dermatofibrosarcoma protuberans

Initial imaging included an ultrasound scan that showed a 2-cm hypoechoic focal lesion at 3 O’clock, 5 cm from the nipple. Subsequent breast MRI showed a well-circumscribed lesion with no deep chest wall invasion or internal mammary and axillary nodal involvement (Figure 1). No further staging imaging was indicated. Ultrasound-guided fine-needle-aspiration showed the lesion was in the subcutaneous tissue of the left breast. It was identified to be a highly cellular spindle cell lesion. A core biopsy of the lesion was performed, which confirmed the lesion to be DFSP.

Surgical excision in the form of mastectomy or wide local excision was recommended, and the patient was referred to breast surgeons. The patient opted for breast-conserving surgery and a left breast wide local excision to remove the DFSP was performed. Superior and inferior margins of more than 20 mm and lateral margins of 22 mm were achieved, but a medial margin of only 5.9 mm was obtained. Since the targeted margin was 20 mm, re-excision was recommended.

**Figure 2:**
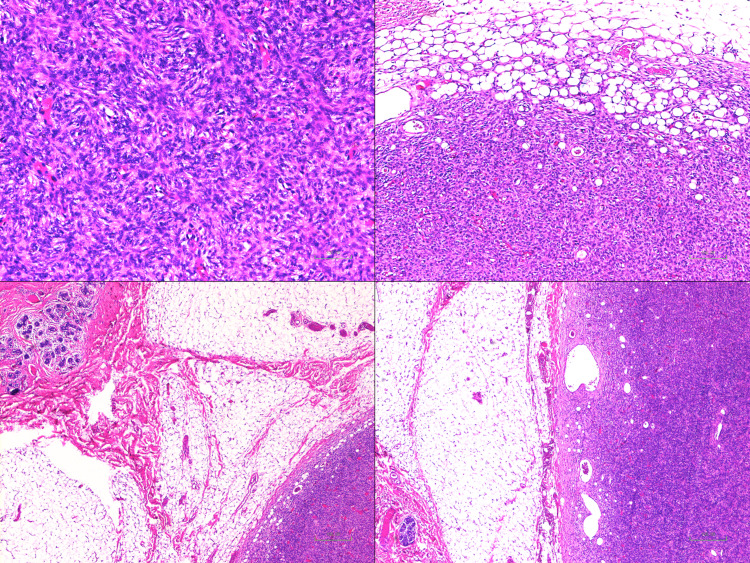
Histological confirmation of DFSP Histology revealed an atypical spindle cell tumour with a storiform appearance involving dermis, subcutaneous fat, and breast parenchyma. The tumour infiltrated adjacent adipose tissue and in some area’s individual fat cells. The tumour did not extend to the deep margin. No epithelial or glandular structures were included in the tumour mass. Adjacent breast tissue showed no evidence of stromal, ductal, or lobular abnormalities DFSP: dermatofibrosarcoma protuberans

**Figure 3:**
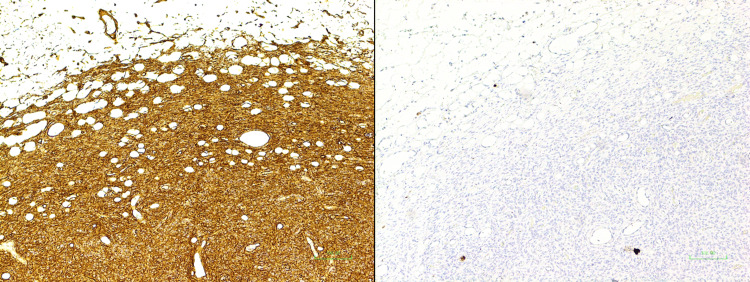
Immunohistochemistry of the DFSP Immunohistochemistry showed negative tumour staining for cytokeratin AE1/3, CK5/6, and high molecular levels with keratin 34 beta E12 and S100. The tumour cells were strongly and diffusely positive for CD34 as shown in the image. These findings support the morphologic diagnostic appearance of DFSP DFSP: dermatofibrosarcoma protuberans

The pathologists indicated that the tumour was small, with a maximum diameter of 13 mm, well-circumscribed, and confined to the subcutis. The specimen had a uniform tan appearance. No involvement of adjacent breast tissue was identified. The remaining fibroadipose tissue appeared unremarkable and lymphovascular invasion was not identified. The histological type was confirmed to be DFSP (Figure 2), with a mitotic rate of <1 per 10 hpf, and immunohistochemistry also showed positive staining for keratin 34 beta E12 (34BE12), S100, and negative staining for cytokeratin AE1/3, CK5/6 (Figure 3). 

The patient was offered options of observation alone, margin re-excision medially with a partial volume reconstruction (after a period of observation), or total mastectomy with immediate reconstruction due to the possibility of poor cosmesis with further breast tissue removal from reoperation. The patient elected to continue with close observation alone due to the chances of poor cosmesis and did not want to proceed with reconstruction with mastectomy. This decision was supported by the slow growth pattern of DFSP, its tendency to recur locally rather than distantly, and its propensity to metastasize only very rarely, in which case, a more aggressive surgical resection could be undertaken. 

The plastic and reconstructive surgeons were consulted during the treatment of this patient and their recommendations were excision with 20-mm margins. A postoperative multidisciplinary team including the breast surgeons, breast radiologist, pathologist, and medical and radiation oncologists were involved in the management of this patient, and the consensus recommended against chemotherapy or radiation therapy with close surgical follow-up. The follow-up recommended for this patient was three monthly clinical reviews and examination with a repeat MRI in one year's time.

## Discussion

DFSP can develop in scars from previous operations, burn wounds, and varicella or Bacillus Calmette-Guérin immunizations, and it grows rapidly during pregnancy [6]. Other less common triggers include trauma, prolonged exposures to arsenic, acanthosis nigricans, and enteropathic acrodermatitis and these have been reported to be associated with DFSP of the breast in 10-20% of cases [6].

The tumours usually present as solitary or clusters of well-circumscribed, reddish-brown, hardened plaques (similar to a keloid) [3,6]. They are initially asymptomatic but can ulcerate, bleed, or become painful. They are most frequently located on the trunk (50-60%), proximal extremities (20%), and head and neck (10-15%) [7]. Lesions progress slowly over months to several decades and eventually develop into rubbery nodules within plaques. Notably, growth accelerates once nodules appear [4]. Lesions usually vary in size, ranging from 1 to 5 cm but some of them can reach up to 20 cm or more [3,8].

There are no recommended excision margins specifically for DFSP of the breast with most previous cases aiming for resection margins of 2-4 cm [9-11] and even up to 5 cm [12]. The National Comprehensive Cancer Network has recommended 2-4-cm resection margins for DFSP but no sub-criteria for DFSP of the breasts have been proposed [13]. Complete surgical removal of DFSP is difficult due to microscopic dissemination of tumour by cell projections under the skin [6]. Both breast-conserving surgery and mastectomy have been reported as viable surgical options, and several factors such as patient history, family history, and patient preference need to be considered when deciding on a definitive treatment [14,15]. Furthermore, neoadjuvant therapy with imatinib has been shown to be effective against DFSP with some cases requiring treatment to allow for breast-conserving surgery to occur [14,16]. Neoadjuvant therapy was not considered in our patient as the lesion was small enough that breast-conserving surgery was not contraindicated initially.

Local recurrence rates can vary from 1% in patients where narrow margins (median of 2 cm) have been achieved to up to 60% in those who have had inadequate surgical margins. Late recurrences (more than 10 years after complete surgical resection) are rare [17-19]. Less than 5% of all cases of DFSP metastasize (4% to distant organs via hematogenous dissemination).

Data regarding follow-up duration and frequency is scarce. Fleury et al. recommend close patient follow-up every three to six months during the initial three years after surgery and yearly reviews indefinitely afterward to identify local recurrences (50-75% occur within three years after excision) and late occurrences (rare) [3,6,19].

## Conclusions

In this report, we have highlighted the difficulties in diagnosing DFSP within the breast, due to an atypical appearance in a patient who had no associated risk factors. A review of the literature revealed that evidence around the management of DFSP within the breast is scarce, in particular, specific guidelines for resection margins for DFSP of the breast, follow-up, and even the role of neoadjuvant or adjuvant therapy. Currently, surgical treatment remains the best approach to manage these tumours. Surgical treatment typically includes either breast-conserving or mastectomy options; however, with expanding reconstructive options, breast-conserving surgical resections with wider margins may become more acceptable in the future.
